# Supplementary material for: Molecular Characterization and SNP-Based Molecular Marker Development of Two Novel High Molecular Weight Glutenin Genes from Triticum spelta L
Source: Int J Mol Sci. 2022 Sep 21;23(19):11104. doi: 10.3390/ijms231911104 (PMC9570065; doi:10.3390/ijms231911104)
Supplement: Supplementary file 1 [file ijms-23-11104-s001.zip › Supplementary material-Tables and Figures Legend.pdf]

Tables and Figures Legend

Figure S1: AS-PCR amplification of HMW-GS genes from spelt wheat cultivars. 1-2: Spelt 137. 3-4: Spelt 6.

Figure S2: Evaluation of the predicted 3D structure of 6 HMW-GSs in wheat. A: Prediction alignment error B: Residual confidence score C: Matching template.

Figure S3: Predictive model for the 3D structure of 6 HMW-GSs in wheat. The 3D protein model confidence score rank. 1Ax2.1*: rank-1 to 5 is listed as 33.9, 31.8, 30.4, 28.6, 29.4; 1Ax1: rank-1 to 5 is listed as 34.9, 31.9, 30.7, 29.9, 28.8; 1Ax2*: rank-1 to 5 is listed as 34.4, 31.8, 30.4, 20.1, 28.6; 1By19*: rank-1 to 5 is listed as 36.9, 33.7, 35.3, 32.0, 31.0; 1By16: rank-1 to 5 is listed as 37.4, 33.6, 34.8, 31.6, 30.8; 1By9: rank-1 to 5 is listed as 37.7, 33.8, 35.7, 32.4, 31.1; 1By8: rank-1 to 5 is listed as 37.2, 33.4, 35.0, 32.4, 31.1; 1By18: rank-1 to 5 is listed as 37.0, 32.8, 34.9, 31.9, 31.1.

Figure S4: PCR amplification of the promoter sequences of *1Ax2.1** (A) and *1By19** (B) from spelt wheat.

Figure S5: Molecular marker primers of *1Ax2.1** gene. The red boxes indicate template of the primer 2.1*F and 2.1*R, green boxes indicate TATA-box (TATA), blue boxes indicate MYC(CAATTG), purple boxes indicate A-box (CCGTCC), black boxes indicate ABRE (ACGTG).

Figure S6: Molecular marker primers of *1By19** gene. The red boxes indicate template of the primer 19*F and 19*R, green boxes indicate AE-box (AGAAACAA), black boxes indicate MYC(CAATTG), purple boxes indicate G-Box (CACGTT).

Table S1: Wheat materials used in the current study and their *Glu-1* compositions.

Table S2: The primer sequences used to amplify HMW-GS genes.

Table S3: Estimation of divergence time (MYA) among 27 HMW-GS genes.

Table S4: The sequences of primers used to amplify the upstream promoter region of HMW-GS genes.

Table S5: Molecular marker primers used for amplifying HMW-GS genes.

Table S6: The amplification conditions for molecular marker development of *1Ax2.1** and *1By19** genes.
