# Supplementary figures and images for: Molecular Characterization and SNP-Based Molecular Marker Development of Two Novel High Molecular Weight Glutenin Genes from Triticum spelta L
Source: Int J Mol Sci. 2022 Sep 21;23(19):11104. doi: 10.3390/ijms231911104 (PMC9570065; doi:10.3390/ijms231911104)

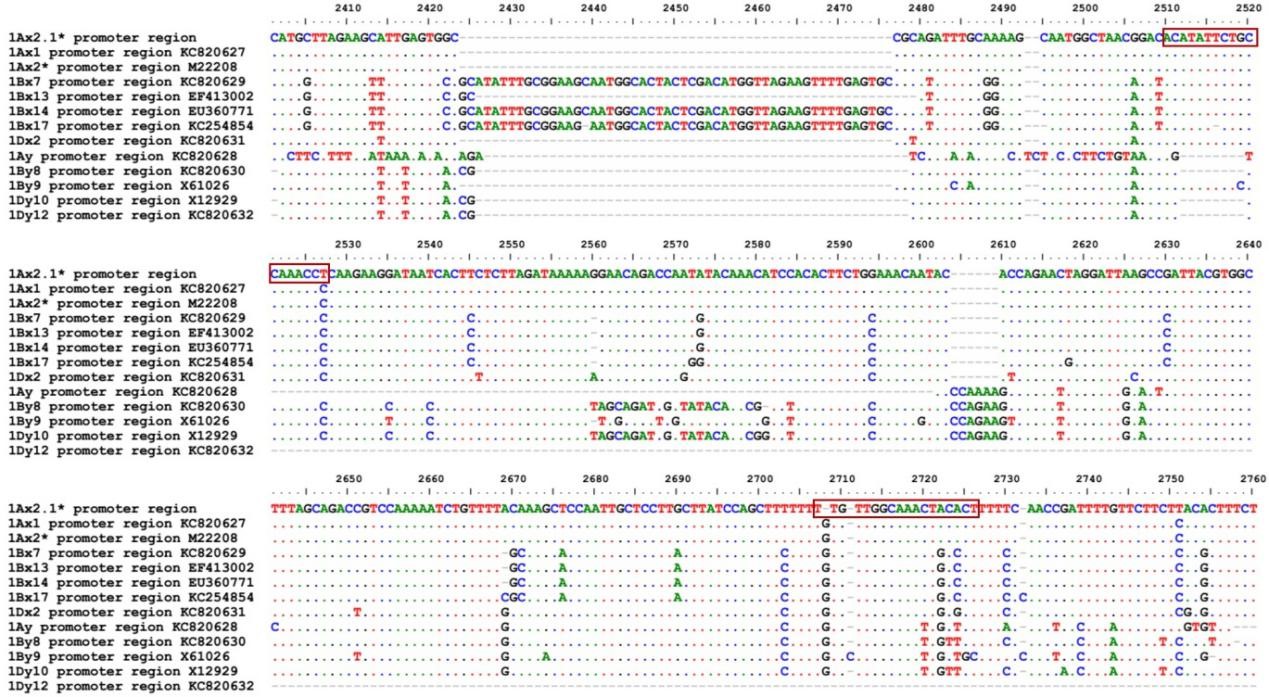

Supplement: Supplementary file 1 [file ijms-23-11104-s001.zip › Figure S5.jpg]

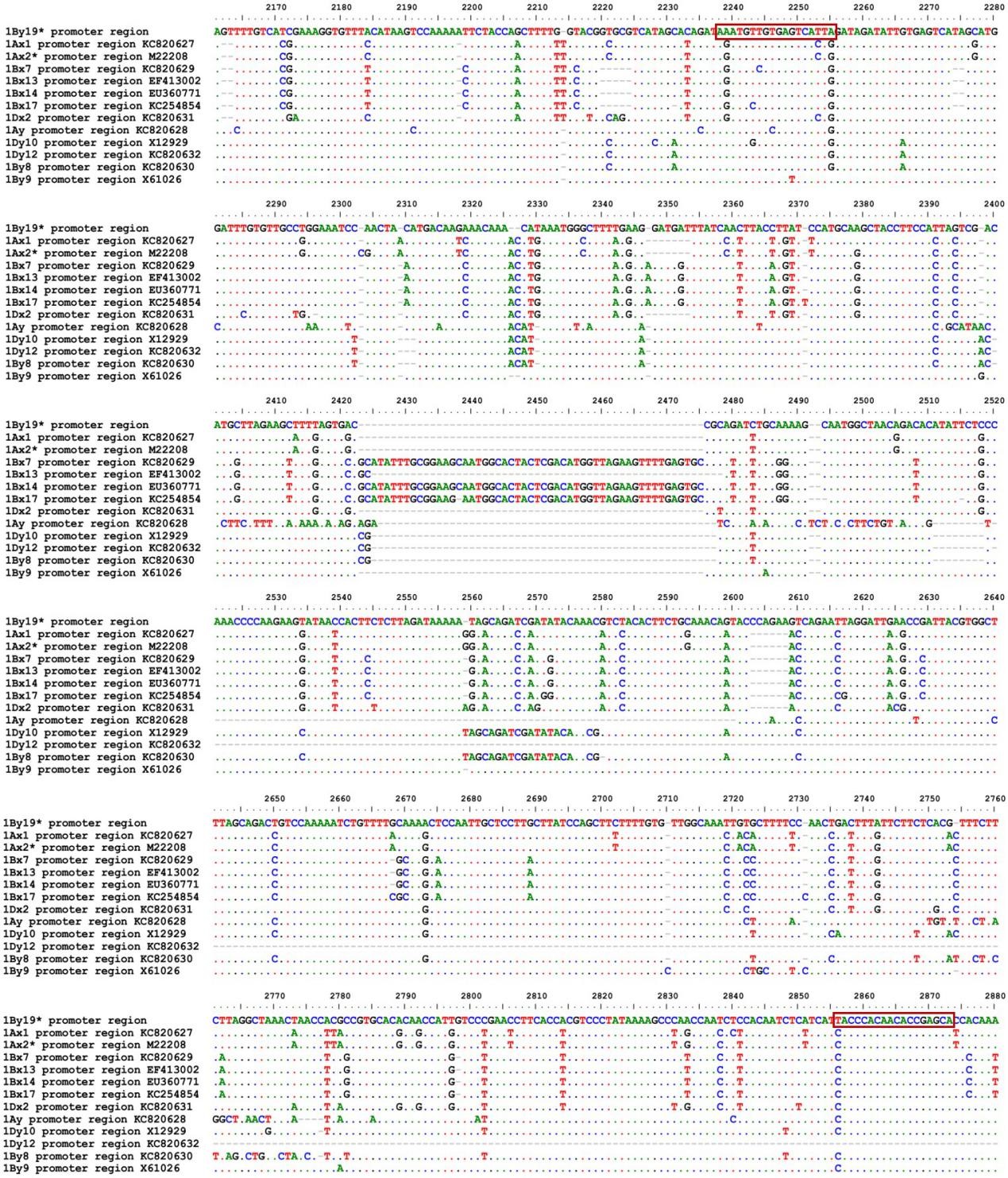

Supplement: Supplementary file 1 [file ijms-23-11104-s001.zip › Figure S6.jpg]

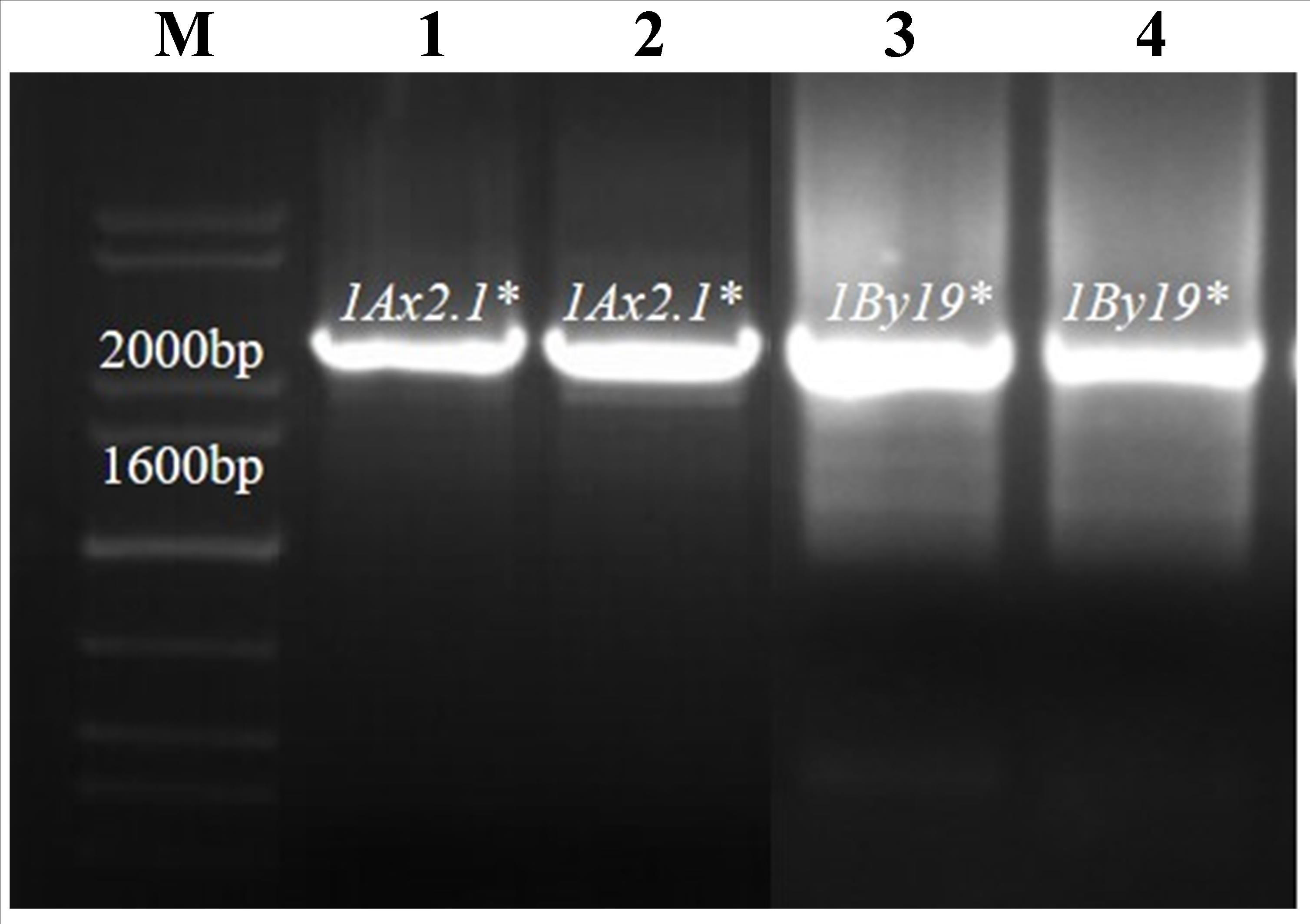

Supplement: Supplementary file 1 [file ijms-23-11104-s001.zip › Figure S1.jpg]

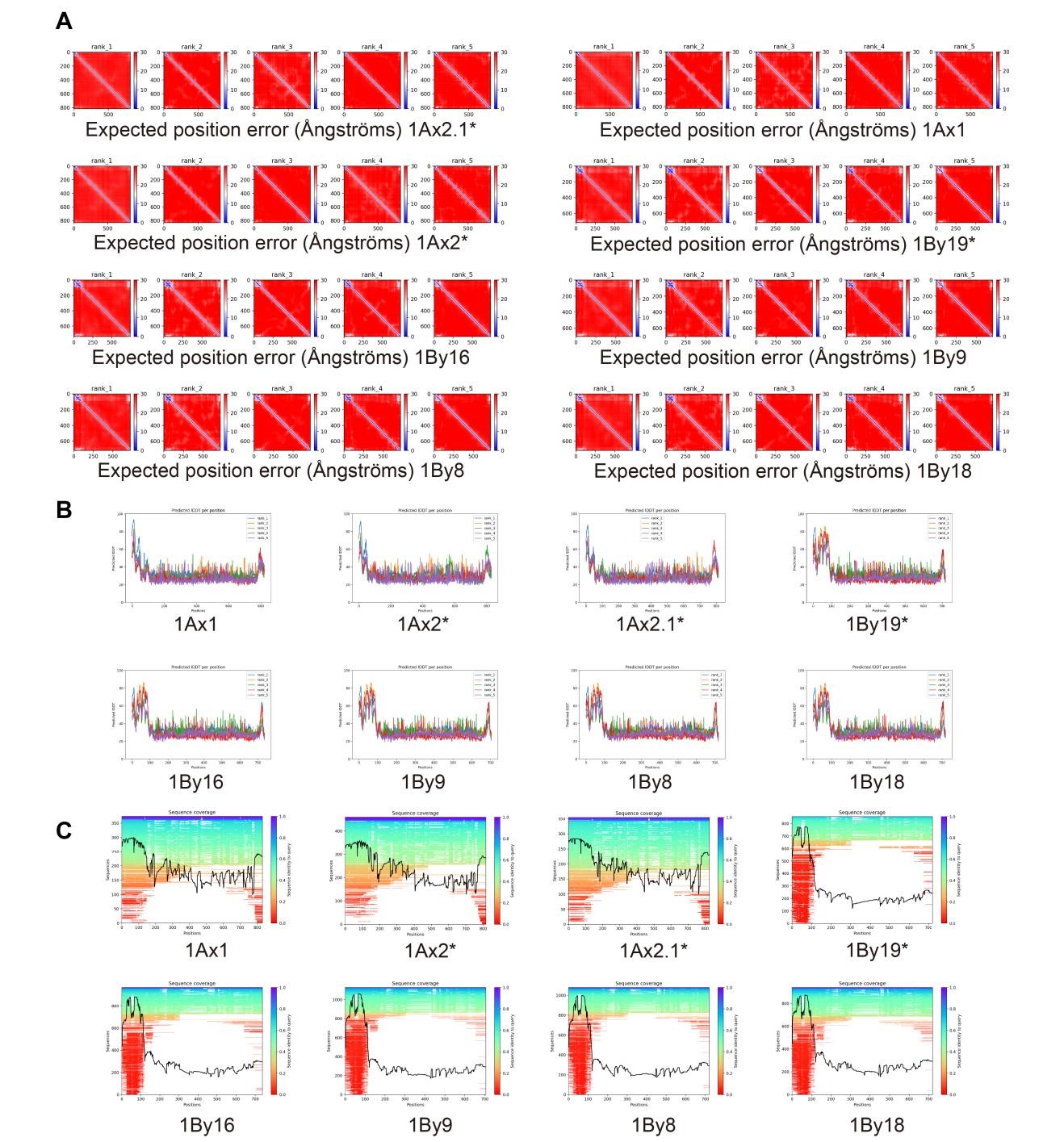

Supplement: Supplementary file 1 [file ijms-23-11104-s001.zip › Figure S2.jpg]

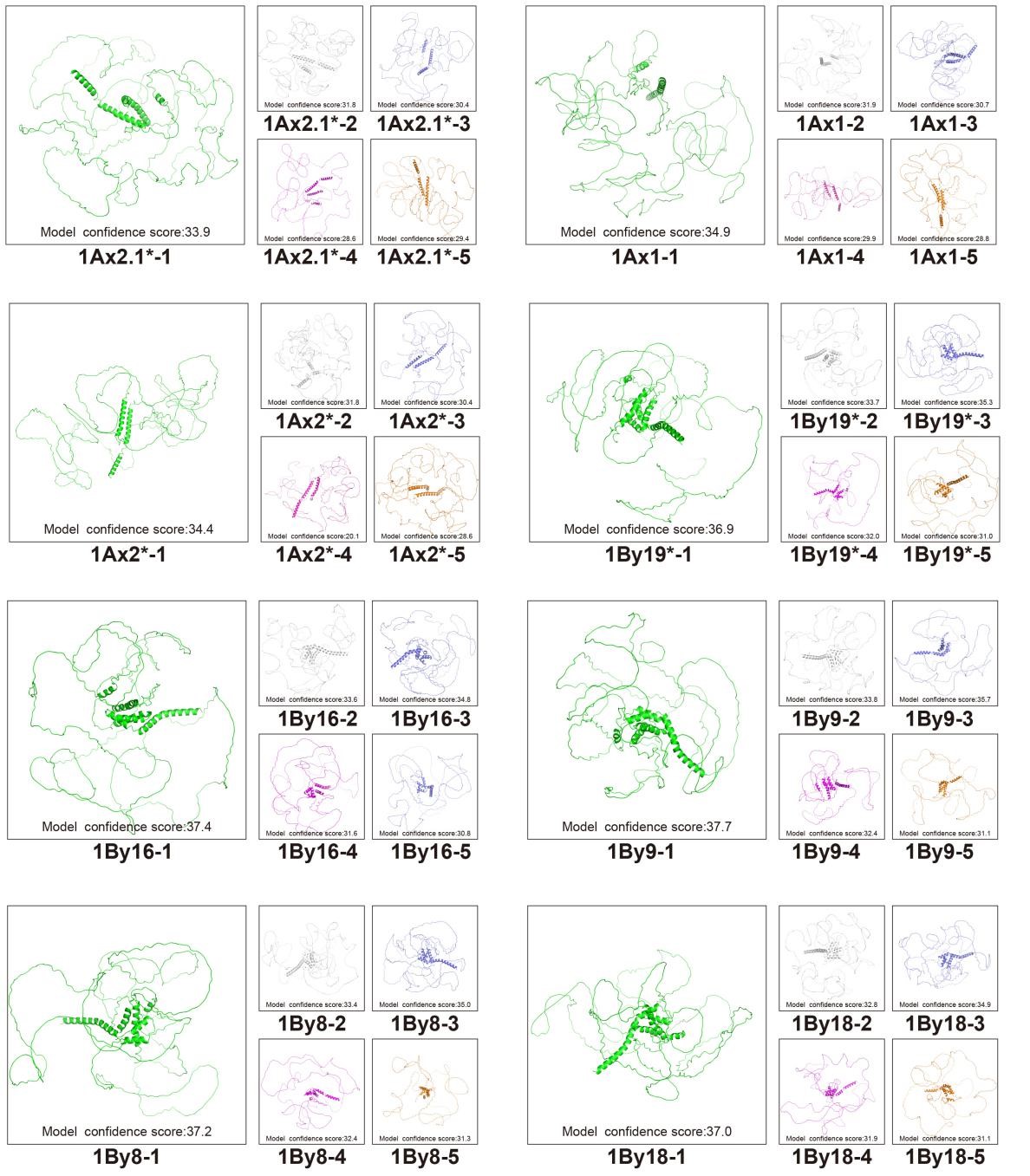

Supplement: Supplementary file 1 [file ijms-23-11104-s001.zip › Figure S3.jpg]

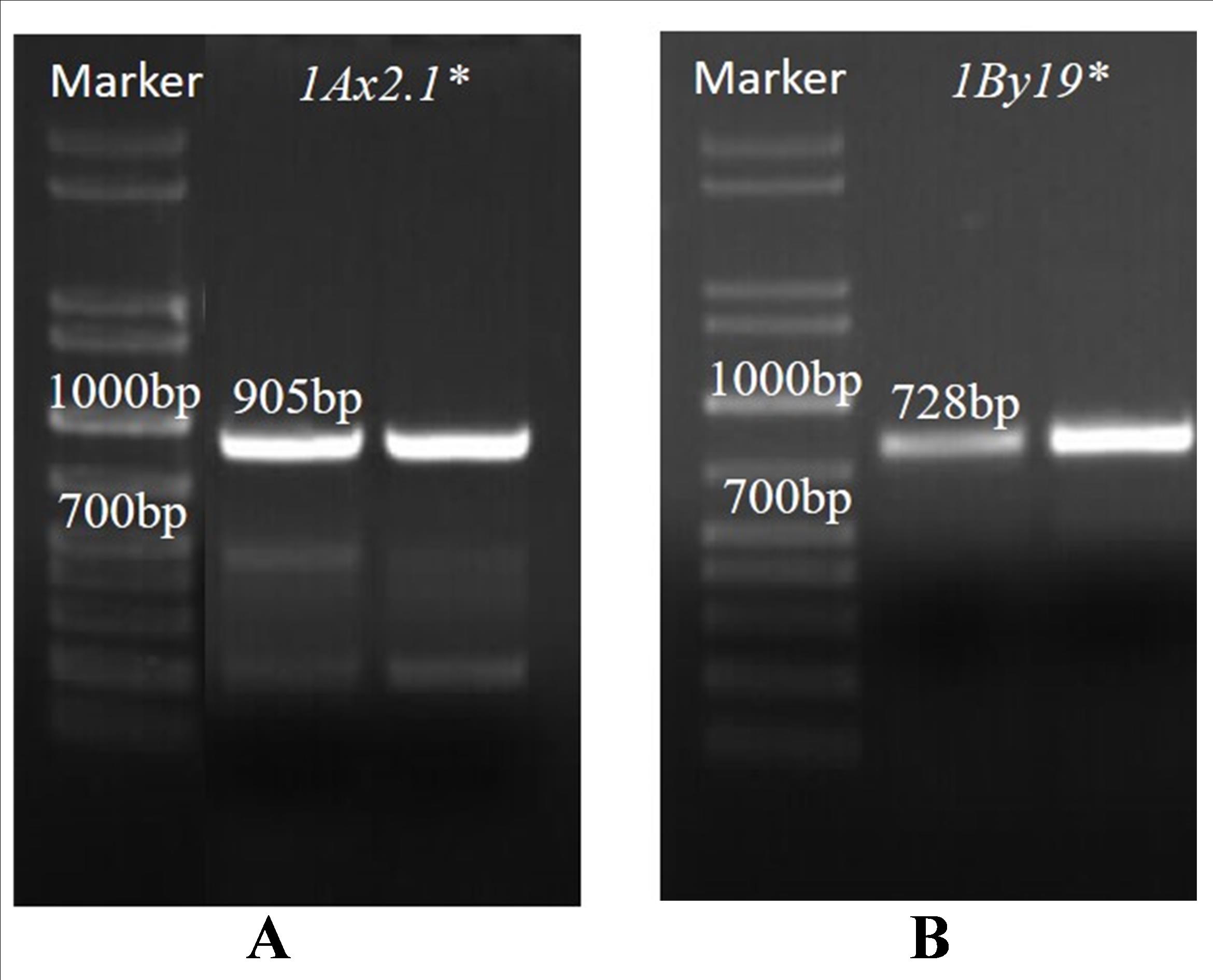

Supplement: Supplementary file 1 [file ijms-23-11104-s001.zip › Figure S4.jpg]
